# Author Correction: SREBP1 drives keratin-80-dependent cytoskeletal changes and invasive behavior in endocrine-resistant ERα breast cancer

**DOI:** 10.1038/s41467-019-11801-w

**Published:** 2019-08-19

**Authors:** Ylenia Perone, Aaron J. Farrugia, Alba Rodríguez-Meira, Balázs Győrffy, Charlotte Ion, Andrea Uggetti, Antonios Chronopoulos, Pasquale Marrazzo, Monica Faronato, Sami Shousha, Claire Davies, Jennifer H. Steel, Naina Patel, Armando del Rio Hernandez, Charles Coombes, Giancarlo Pruneri, Adrian Lim, Fernando Calvo, Luca Magnani

**Affiliations:** 1 Department of Surgery and Cancer, Imperial College London, London, UK; 2 Division of Cancer Biology, Tumour Microenvironment Team, Institute of Cancer Research, London, UK; 3 MTA TTK Lendület Cancer Biomarker Research Group, Institute of Enzymology, Hungarian Academy of Sciences, Budapest, Hungary; 4 2nd Department of Pediatrics, Semmelweis University, Budapest, Hungary; 5 European Institute of Oncology, Milan, Italy; 6 Faculty of Engineering, Department of Bioengineering, Imperial College London, London, UK; 7 Department for Life Quality Studies, Alma Mater Studiorum, University of Bologna, Rimini, Italy; 8 Department of Chemistry, Imperial College London, London, UK; 9 Histopathology Department, Imperial College London, Charing Cross Hospital NHS Trust, London, UK; 10 ECMC Imperial College. Department of Surgery and Cancer, Imperial College London, London, UK; 11 Pathology Department, Fondazione IRCCS Istituto Nazionale Tumori and University of Milan, School of Medicine, Milan, Italy; 12 Present Address: MRC Molecular Haematology Unit, Haematopoietic Stem Cell Biology Laboratory, Weatherall Institute of Molecular Medicine, University of Oxford, Oxford, UK; 13 Present Address: Instituto de Biomedicina y Biotecnologia de Cantabria, Santander, Spain

**Keywords:** Breast cancer, Cytoskeleton, Mechanisms of disease, Histone post-translational modifications, Epigenetics

Correction to: *Nature Communications* 10.1038/s41467-019-09676-y, published online 9 May 2019.

This Article contained an error in the name of the author Alba Rodríguez-Meira, which was incorrectly given as Alba Rodríguez Meira.

These errors have been corrected in the PDF and HTML versions of the article.

