# Supplementary material for: SREBP1 drives Keratin-80-dependent cytoskeletal changes and invasive behavior in endocrine-resistant ERα breast cancer
Source: Nat Commun. 2019 May 9;10:2115. doi: 10.1038/s41467-019-09676-y (PMC6509342; doi:10.1038/s41467-019-09676-y)
Supplement: Supplementary file 1 — Supplementary Information [file 41467_2019_9676_MOESM1_ESM.docx]

**SREBP1 drives Keratin 80-dependent cytoskeletal changes and invasive behavior in endocrine resistant ERα breast cancer**

Perone Y et al.

**Supplementary Information**

**Supplementary Data 1: KRT80 expression in BC cell lines.** Fifty-one breast cancer cell lines, including estrogen receptor positive and negative lines, were assessed for KRT80 expression by meta-analysis of previously published microarray datasets (probe 231849_at). Cells over-expressing KRT80 are in orange, while cells under-expressing KRT80 are in blue. Over and under-expression has been calculated over the normalized median value for the entire expression dataset.

**Supplementary Data 2: RNA-seq analysis of KRT80 overexpression in MCF7 cells.** All significantly up-regulated genes (Fold Change >2, qValue<0.01) are included. The second tab includes additional gene ontology analysis (http://software.broadinstitute.org/gsea/msigdb/annotate.jsp).

**Supplementary Figure 1: KRT80 expression in BC cell lines and Breast Cancer.** A) Targeted RT-qPCR for genes that were found differentially regulated in RNA-seq analysis. Bars and lines represent mean and SD of three independent measurements. B) KRT80 transcripts were also quantified in non-tumorigenic breast MCF10A cell lines and in drug sensitive and drug-resistant clones of T47D breast cancer cell lines. Bars and error bars represent the average and the SD of 3 biological replicates. One-Way ANOVA with Dunnet’s correction was used to establish statistical significance. Asterisks represent significance levels at *, **, *** P<0.05, 0.01 and 0.001 respectively. C) KRT80 expression in diagnostic material has prognostic significance. Analysis were performed on a meta-collection of batch-normalized microarray from GEO browser. Patients were split into high and low KRT80 expression. All histologies were included in the analysis. D) Multivariate correction analysis for panel C. E) KRT80 expression in diagnostic material has prognostic significance in the context of post-progression survival (PPS), distal-metastasis free survival (DMFS) and overall survival (OS). Number of patients in each arm is indicated. Hazard ratios with confidence intervals and univariate p-values are also shown.

**Supplementary Figure 2: KRT80 expression in Breast Cancer patients.**

A) Kaplan-Meier analysis using KRT80 expression levels in a large independent cohort of ERα breast cancer patients (TCGA) profiled with RNA-seq. B) KRT80 prognostic significance was investigated in three sub cohorts derived from the METABRIC cohorts and were tested in function of prognostic power in the short-term (<5 years) or long-term (<25years). Hazard ratios are plotted on the x-axis (>1= worse overall survival). C) KRT80 prognostic association with overall survival was tested in patients which had annotated post-surgical adjuvant treatment.

**Supplementary Figure 3: KRT80 enhancer mapping.** A) Enhancer ranking strategy was applied to all the individual subpeaks within the original 12.5Kb E1. Individual sub- regions have distinct predicted clonality with the core E1 enhancer consistently showing higher clonality. Cells characterized by KRT80 expression have consistent higher stronger predicted clonality for the E1 enhancer. Topology for the E1 enhancer locus with associated DHS-seq peaks is shown in the right inset. B) Virtual 4C (<http://promoter.bx.psu.edu/hi-c/virtual4c.php>) using POL2 Chia-PET data to predict potential loops between the E1 and E2 loci and KRT80 promoter in MCF7 cells. C) Enhancer ranking strategy was applied to all available H3K27ac ChIP-seq dataset from the ENCODE project to predict KRT80 subpopulations in various tissues and cell lines. D) RNA levels for KRT80 in a large panel of cell lines. Dotted lines represent changes over the sample-wide normalized median at 5-fold, 10-fold and 100-fold.

**Supplementary Figure 4: SREBP1 is recruited at E1 locus in KRT80-positive models.** A) The 12.5Kb E1 contains distinct sub-enhancers with the core 1.5Kb showing the highest correlation with KRT80 expression. ChIP-seq for SREBP1 in several tissue for the E1 enhancer is shown. KRT80 positive lung cancer A549 are displayed in dark red. B) RNA-seq profiles from ENCODE cell lines around the KRT80 locus and E1 enhancer locus. KRT80 positive lung cancer A549 are displayed in blue. The middle panel shows a zoomed area with overlaid the E1 topology derived from breast cancer cell lines. Normalized RPKM are shown in the scale. KRT80 positive HTC116 are shown for comparison. SREBP1 ChIP-seq signal in A549 is shown in dark red. The bottom panel shows SREBP1 expression and SREBP1 binding at the SREBP1 locus for comparison. SREBP1 binding at SREBP1 promoter serves as a positive control for SREBP1 ChIP. C) ChIP-seq signal at LINC00263 and KRT80 E1 enhancer is shown for T47D parental and AI resistant T47D-LTED breast cancer cells. D) Metanalysis of KRT80 transcriptional correlation with SREBP1 canonical targets in ERα breast cancer samples.

**Supplementary Figure 5. E1 is not evolutionary conserved.** Conservation analysis in the E1 locus and nearby area.

**Supplementary Figure 6. KRT80 mRNA expression in normal breast.** Meta-analysis of TCGA data and Affymetrix GEO data comparing expression levels for KRT80 in normal samples and cancer samples.

**Supplementary Figure 7. Additional validation of KRT80 antibodies.** Representative images of IHC using two independent antibodies for KRT80 in breast tissues and breast cancer samples. Antibody are labelled accordingly and were obtained from the Protein Atlas Initiative. Bottom four panels show actual samples from the prospective trial in which Shearwave Elastography was conducted.

**Supplementary Figure 8. Functional characterization of KRT80.** A) RT-qPCR measurements of KRT80 mRNA levels in breast cancer cells transfected with KRT80-DKK constructs. B) Western Blot analysis of KRT80 protein levels in breast cancer cells transfected with KRT80-DKK constructs. Both KRT80 and DKK tagged are shown. C) RT-qPCR and Western Blot analysis of KRT80 mRNA and protein in cells transfected with stable shKRT80 constructs. D) RT-qPCR and Western Blot analysis of KRT80 mRNA and protein in cells transfected with transient siKRT80 constructs. E) Migration analysis of transiently transfected siRNA-KRT80 cells on 2D surface. F) Matrigel invasion analysis of transiently transfected siRNA-KRT80. Invasion and Migration experiments using transient siRNA were conducted in biological triplicates using invasive and migratory LTED cell in which KRT80 is highly expressed. Bars and error bars represent the average and the SD of 3 biological replicates. One-Way ANOVA with Dunnet’s correction was used to establish statistical significance. Asterisks represent significance levels at *, **, *** P<0.05, 0.01 and 0.001 respectively.

**Supplementary Figure 9. KRT80 is preferentially distributed at the margin of 3D cultures.** A) IF analysis of KRT80 in MCF7 cells and drug-resistant derivatives. B) IF imaging of KRT80 in breast cancer cell lines grown in 3D spheroids. Blown up of spheroids margins are shown in the right panels C) IF analysis of KRT80 in cancer cells isolated from fresh pleural effusion from breast cancer patients.
