# Supplementary material for: SREBP1 drives Keratin-80-dependent cytoskeletal changes and invasive behavior in endocrine-resistant ERα breast cancer
Source: Nat Commun. 2019 May 9;10:2115. doi: 10.1038/s41467-019-09676-y (PMC6509342; doi:10.1038/s41467-019-09676-y)
Supplement: Supplementary file 3 — Description of Additional Supplementary Files [file 41467_2019_9676_MOESM3_ESM.docx]

**Description of Supplementary Files**

**File Name:** Supplementary Data 1

**Description:** KRT80 expression in BC cell lines. Fifty-one breast cancer cell lines, including estrogen receptor positive and negative lines, were assessed for KRT80 expression by meta-analysis of previously published microarray datasets (probe 231849_at). Cells over-expressing KRT80 are in orange, while cells under-expressing KRT80 are in blue. Over and under-expression has been calculated over the normalized median value for the entire expression dataset.

**File Name:** Supplementary Data 2

**Description:** RNA-seq analysis of KRT80 overexpression in MCF7 cells. All significantly up-regulated genes (Fold Change >2, qValue<0.01) are included. The second tab includes additional gene ontology analysis (http://software.broadinstitute.org/gsea/msigdb/annotate.jsp).
